# Microcirculatory effects of the transfusion of leukodepleted or non-leukodepleted red blood cells in patients with sepsis: a pilot study

**DOI:** 10.1186/cc13730

**Published:** 2014-02-17

**Authors:** Abele Donati, Elisa Damiani, Michele Maria Luchetti, Roberta Domizi, Claudia Scorcella, Andrea Carsetti, Vincenzo Gabbanelli, Paola Carletti, Rosella Bencivenga, Hans Vink, Erica Adrario, Michael Piagnerelli, Armando Gabrielli, Paolo Pelaia, Can Ince

**Affiliations:** 1 Department of Biomedical Sciences and Public Health, Anesthesia and 640 Intensive Care Unit, Università Politecnica delle Marche, via Tronto 10/a, 60020 Torrette di Ancona, Italy; 2 Anesthesia and Intensive Care Unit, Azienda Ospedaliera Universitaria “Ospedali Riuniti”, via Conca 71, 60126 Torrette di Ancona, Italy; 3 Department of Translational Physiology, Academic Medical Center, Meibergdreef 9, 1105 AZ Amsterdam, The Netherlands; 4 Immunohematology and Transfusional Medicine, Azienda Ospedaliera Universitaria “Ospedali Riuniti”, via Conca 71, 60126 Torrette di Ancona, Italy; 5 Department of Physiology, Cardiovascular Research Institute Maastricht, Maastricht University, P.O. Box 616, 6200 MD Maastricht, The Netherlands; 6 Department of Intensive Care, CHU-Charleroi, Université Libre de Bruxelles, 808 Route de Lennik, 1070 Brussels, Belgium; 7 Department of Clinical and Molecular Sciences, Clinica Medica, Università Politecnica delle Marche, cia Tronto 10/a, 60020 Torrette di Ancona, Italy

## Abstract

**Introduction:**

Microvascular alterations impair tissue oxygenation during sepsis. A red blood cell (RBC) transfusion increases oxygen (O_2_) delivery but rarely improves tissue O_2_ uptake in patients with sepsis. Possible causes include RBC alterations due to prolonged storage or residual leukocyte-derived inflammatory mediators. The aim of this study was to compare the effects of two types of transfused RBCs on microcirculation in patients with sepsis.

**Methods:**

In a prospective randomized trial, 20 patients with sepsis were divided into two separate groups and received either non-leukodepleted (n = 10) or leukodepleted (n = 10) RBC transfusions. Microvascular density and perfusion were assessed with sidestream dark field (SDF) imaging sublingually, before and 1 hour after transfusions. Thenar tissue O_2_ saturation (StO_2_) and tissue hemoglobin index (THI) were determined with near-infrared spectroscopy, and a vascular occlusion test was performed. The microcirculatory perfused boundary region was assessed in SDF images as an index of glycocalyx damage, and glycocalyx compounds (syndecan-1, hyaluronan, and heparan sulfate) were measured in the serum.

**Results:**

No differences were observed in microvascular parameters at baseline and after transfusion between the groups, except for the proportion of perfused vessels (PPV) and blood flow velocity, which were higher after transfusion in the leukodepleted group. Microvascular flow index in small vessels (MFI) and blood flow velocity exhibited different responses to transfusion between the two groups (*P* = 0.03 and *P* = 0.04, respectively), with a positive effect of leukodepleted RBCs. When within-group changes were examined, microcirculatory improvement was observed only in patients who received leukodepleted RBC transfusion as suggested by the increase in De Backer score (*P* = 0.02), perfused vessel density (*P* = 0.04), PPV (*P* = 0.01), and MFI (*P* = 0.04). Blood flow velocity decreased in the non-leukodepleted group (*P* = 0.03). THI and StO_2_ upslope increased in both groups. StO_2_ and StO_2_ downslope increased in patients who received non-leukodepleted RBC transfusions. Syndecan-1 increased after the transfusion of non-leukodepleted RBCs (*P* = 0.03).

**Conclusions:**

This study does not show a clear superiority of leukodepleted over non-leukodepleted RBC transfusions on microvascular perfusion in patients with sepsis, although it suggests a more favorable effect of leukodepleted RBCs on microcirculatory convective flow. Further studies are needed to confirm these findings.

**Trial registration:**

ClinicalTrials.gov, NCT01584999

## Introduction

Anemia is a common problem in intensive care units (ICUs) [1]. Red blood cell (RBC) transfusion aimed at increasing O_2_ delivery is generally considered a life-saving treatment [2]. Nonetheless, a restrictive transfusion strategy may result in equivalent or improved clinical outcomes in comparison with liberal transfusion strategies [3]. This raises concern whether transfusion practice is beneficial in general.

Altered properties of stored blood products might explain this paradoxical effect. Biochemical and hemorrheological alterations of packed RBC units due to prolonged storage (depletion of ATP and 2,3-diphosphoglycerate, membrane phospholipid vesiculation and loss, protein and lipid peroxidation, and loss of deformability) [4-6] may affect their O_2_ delivery capacity [7-9]. Residual leukocytes in RBC units may also compromise the efficacy of blood transfusion by producing cytokines such as interleukin (IL)-1b, IL6, IL8, and tumor necrosis factor-alpha (TNFα), which may interfere with immune function [10], alter circulating lymphocytes, and enhance neutrophil activity in recipients [11,12]. These effects, collectively referred to as “transfusion-related immunomodulation”, may be responsible for higher incidences of infections among transfused patients [13]. Moreover, cytokines may contribute to RBC membrane alterations during storage [14] and impair RBC rheology. These effects, in theory, should be prevented by leukocyte reduction; however, clinical data remain controversial [15].

The effects of blood transfusion in patients with sepsis, and in particular how it influences tissue microcirculation, are poorly understood. Sepsis-induced microvascular dysfunction [16], endothelial and glycocalyx damage [17], and pathological shunting and heterogeneous perfusion [18] may hamper blood transfusion-based restoration of tissue RBC delivery and oxygenation [19]. Moreover, altered RBCs or inflammatory mediators in blood bags (or both) may further compromise microvascular perfusion and tissue O_2_ uptake. Only a few studies have previously investigated the response of the microcirculation to blood transfusion in septic populations [20-22] and found no global effect on microvascular perfusion; unfortunately, however, the characteristics of the transfused RBCs in these studies were not examined. The primary aim of the present study was to compare the effects of non-leukodepleted or leukodepleted RBC transfusions on microvascular flow in patients with sepsis. In addition, it was determined whether transfusion of either non-leukodepleted or leukodepleted packed RBC units could increase microcirculatory density and reactivity to improve tissue oxygenation during sepsis.

## Materials and methods

The study protocol was approved by the local medical ethics committee of the Azienda Ospedaliero Universitaria (AOU) Ospedali Riuniti of Ancona in Italy (NCT01584999, www.clinicaltrials.gov). Written informed consent was obtained from the enrolled patients or their next of kin.

### Patients

Between February 2011 and 2012, adult patients admitted to the 12-bed ICU of the AOU Ospedali Riuniti of Ancona with sepsis, severe sepsis, or septic shock as diagnosed according to standard criteria [23] and requiring blood transfusion for hemoglobin (Hb) levels of less than 8 g/dL or as indicated by the attending physician (in accordance with the local hospital guidelines) were eligible to participate in this prospective randomized study. Exclusion criteria for this study were age of less than 18 years, previous blood transfusions during ICU stay, previous history of coagulation disorders, cardiogenic or hemorrhagic shock, pregnancy, and factors impeding the sublingual microcirculation evaluation (oral surgery and maxillofacial trauma). All patients were monitored with an arterial catheter. Sedation and analgesia were provided according to individual needs, as well as the type of fluids infused (crystalloids and colloids) and adrenergic agents (norepinephrine and dobutamine). The goal was to maintain a mean blood pressure of 65 mm Hg as recommended by the international guidelines of the Surviving Sepsis Campaign (2008) [24]. Fluid infusion and furosemide treatment were titrated according to individual needs in order to maintain adequate urine output (>0.5 mL/kg per hour) [24].

### Interventions

The original study protocol included three separate groups of septic patients receiving transfusion of fresh (<10 days of storage) non-leukodepleted, fresh leukodepleted, or old (>15 days of storage) non-leukodepleted RBC units, respectively. The analysis presented herein was focused on the role of leukocyte reduction; therefore, only the data from the first two groups (hereafter referred to as non-leukodepleted and leukodepleted groups) are reported. A parallel analysis focused on the role of prolonged storage will be reported separately. Blood product randomization was performed through sealed envelopes by a physician at the blood bank, who blindly provided the blood bags to the ICU; neither the attending physician nor the investigators nor the patients were aware of the type of RBCs transfused. Post-storage leukoreduction was performed by a blood bank physician using the filter Sepacell RZ-200 (Fenwal, Inc., Lake Zurich, IL, USA) within a maximum of 5 days after donor blood withdrawal.

### Basic hemodynamic and blood gas parameters

All measurements were performed immediately before and 1 hour after RBC transfusions. These time points were chosen on the basis of those reported in previous studies [20-22]. We recorded temperature, heart rate, and mean arterial pressure (MAP). Arterial blood samples were withdrawn in order to assess Hb level, whole blood cell counts, blood gases—pH, arterial partial pressure of oxygen (paO_2_), arterial partial pressure of carbon dioxide (paCO_2_), oxygen saturation (SaO_2_), paO_2_/fraction of inspired oxygen (paO_2_/FiO_2_), HCO_3_^−^, base excess—and lactate (Lac), creatinine, and glucose levels. Arterial blood samples were immediately centrifuged, and plasma and serum were stored at −70°C for subsequent analysis. For each participant, the Simplified Acute Physiology Score (SAPS) II was obtained at admission and the Sequential Organ Failure Assessment (SOFA) score [25] on the study day.

### Microcirculation measurements with sidestream dark field imaging

Sublingual microcirculatory density and flow were monitored by using sidestream dark field (SDF) imaging (Microscan; Microvision Medical BV, Amsterdam, The Netherlands); details on the SDF imaging technique have been described elsewhere [26]. Briefly, the Microscan is a hand-held video microscope system that epi-illuminates a tissue of interest with stroboscopic green (530 nm) light-emitting diodes. Hb absorbs the 530 nm wavelength light, which in turn is captured via the imaging probe’s light guide and a charge-coupled device camera. Clear images of flowing RBCs are depicted as dark moving globules in the lumen of blood vessels against a white/grayish background. After the removal of saliva and other secretions with a gauze, the SDF probe, covered by a sterile disposable cap, was gently applied on the sublingual mucosa of the floor of the mouth at the base of the tongue. Videos from five different sites (at least 10 seconds per site) were recorded at both time points with adequate focus and contrast, and every effort was made to avoid movement and pressure artefacts. Poor-quality images were discarded, and three images for each time point were selected and analyzed by using a computer software package (Automated Vascular Analysis Software; Microvision Medical BV). According to the consensus report on the performance and evaluation of microcirculation using SDF imaging [27], total vessel density (TVD) and perfused vessel density (PVD) were calculated for small vessels (diameter of less than 20 μm). The De Backer score was calculated as described previously [27]. In brief, the SDF image was divided by three equidistant horizontal and three equidistant vertical lines; the De Backer Score was calculated as the number of the small (diameter of less than 20 μm) and medium (diameter of 20 to 100 μm) vessels crossing the lines divided by the total length of the lines. The proportion of perfused vessels (PPV) and the microvascular flow index (MFI), reflecting microcirculatory blood flow velocity, were analyzed semi-quantitatively in small vessels, as described elsewhere [28]. The flow heterogeneity index was also calculated as the highest MFI minus the lowest MFI, divided by the mean MFI, providing an index of heterogeneous microcirculatory perfusion. Quantitative blood flow velocity was measured through the use of space-time diagrams [29]. Three lines were manually traced in the space-time diagram, and the average orientation was used to calculate the blood flow velocity [30].

### Peripheral O_2_ and hemoglobin measurements with near-infrared spectroscopy

Near-infrared reflectance spectrophotometry (InSpectra™ Model 650; Hutchinson Technology Inc., Hutchinson, MN, USA) was used to measure peripheral tissue oxygen saturation (StO_2_) and tissue Hb index (THI) [31,32] at baseline and during a vascular occlusion test (VOT). A 15 mm-sized probe was placed on the skin of the thenar eminence, and a sphygmomanometer cuff was placed around the (upper) arm to occlude the brachial artery. After a 3-minute period of StO_2_ signal stabilization, arterial inflow was arrested by inflation of the cuff to 50 mm Hg above the systolic arterial pressure. The cuff was kept inflated until the StO_2_ decreased to 40% and then released [33]. StO_2_ was continuously recorded during the reperfusion phase until stabilization [33]. The StO_2_ downslope (%/minute) was calculated from the regression line of the first minute of StO_2_ decay after occlusion, providing an index of O_2_ consumption rate. The StO_2_ upslope (%/minute) was obtained from the regression line of StO_2_ increase in the reperfusion phase. The area under the curve (AUC) of the hyperemic response was also calculated. StO_2_ upslope and the AUC of the StO_2_ reflect microvascular reactivity [33]. All the parameters were calculated by using a computer software package (version 3.03 InSpectra Analysis Program; Hutchinson Technology Inc.).

### Microvascular glycocalyx assessment

A series of 10 Microscan video fragments of at least 40 consecutive frames were automatically analyzed by using the GlycoCheck ICU software package (Maastricht University Medical Center, Maastricht, The Netherlands) in order to measure vascular lumen perfused boundary region (PBR). The PBR includes the dimension of the permeable part of the endothelial glycocalyx which allows the penetration of flowing RBCs. Erythrocytes usually have limited access into an intact glycocalyx; when this is compromised and starts losing its protective capacity, its permeability increases, allowing circulating cells to approximate the luminal endothelial membrane. As a result, the dimension of the erythrocyte PBR will increase [34]. This methodology has been extensively described elsewhere [35]. Briefly, measurement lines perpendicular to the vessel direction are arranged automatically every 10 μm along each visible vessel with a diameter of less than 50 μm. Each line represents a measurement site; at each measurement site, 21 parallel (every 0.5 μm) intensity profiles are plotted, and RBC column width (full width half maximum) is determined at each line for all 40 consecutive frames in a movie, revealing a total of 840 RBC column width measurements at a measurement site (21 profiles × 40 frames). The associated (cumulative) distribution of the RBC column widths for these 840 measurements was used to determine median RBC column width (P50) as well as lower and upper percentiles of the RBC column width distribution. The RBC perfused diameter (position of the outer edge of the RBC perfused lumen) is derived from the RBC column width distribution by linear extrapolation of all RBC column width percentiles between P25 and P75. The PBR is defined as the distance of median (P50) RBC column width to the outer edge of the extrapolated RBC perfused diameter.

### Serum measurements of glycocalyx damage markers

Concentrations of syndecan-1 (Human sCD138/Syndecan-1 enzyme-linked immunosorbent assay [ELISA] Gen-Probe Diaclone SAS, Besancon, France), heparan sulfate (Human heparan sulfate HS ELISA Kit; Cusabio Biotech Co., Ltd., Wuhan, Hubei Province 430206, China), and hyaluronan (Hyaluronic Acid Quantitative Test kit; Corgenix, Inc., Broomfield, CO, USA), three main components of the endothelial glycocalyx [36], were measured in serum by using the corresponding ELISA kits in accordance with the instructions of the manufacturer.

### Sample size calculation

Sample size calculation was computed on the basis of MFI data. A total of nine patients per group was shown to be sufficient to detect a statistically significant change in MFI of 0.4 (standard deviation = 0.3) after blood transfusion with a power of 80% and an alpha error of 0.05.

### Statistical analysis

Statistical analysis was performed by using GraphPad Prism version 5 (GraphPad Software, La Jolla, CA, USA). A Mann-Whitney *U* test was used to evaluate differences between the two groups at baseline and after blood transfusion. Wilcoxon matched-pairs signed-rank test was used for comparative analysis of data sets obtained before and 1 hour after RBC transfusion. A Spearman coefficient was evaluated to study the correlation between variables. All data are presented as median (25th-75th percentiles). Differences were considered significant at *P* values of less than 0.05.

## Results

Twenty patients were enrolled in the study (10 patients per group). Patient characteristics are presented in Table 1. All patients were mechanically ventilated. All patients received 2 (2 to 3) packed RBC units, and all transfused RBCs were fresh: median ages were 4 (3.5 to 5) days for non-leukodepleted and 3 (1.5 to 3) days for leukodepleted RBCs.

**Table 1:** Patient characteristics for the two groups

	**Non-leukodepleted group**	**Leukodepleted group**
	**n = 10**	**n = 10**
Age, years	70 (65–72)	74 (64–79)
Sex, male; female	5; 5	7; 3
SAPS II on admission	37 (28–74)	41 (35–47)
ICU days before enrollment	9 (8–12)	9 (4–29)
Sepsis, number	2	5
Severe sepsis, number	3	2
Septic shock, number	5	3
Source of infection, number		
Lung	4	3
Abdomen	1	3
Urinary tract	2	1
Miscellaneous	3	3
Adrenergic dose^a^		
Norepinephrine	5; 0.047 (0.015–0.370)	3; 0.155 (0.050–0.260)
Dobutamine	1; 2.074 (2.074–2.074)	1; 2.31 (2.31–2.31)

^a^Number of patients; median dose in μg/kg*min (interquartile range). ICU, intensive care unit; SAPS II, Simplified Acute Physiology Score II.

### Sequential Organ Failure Assessment score, hematologic, hemodynamic, and gas exchange variables

SOFA score, hematologic, hemodynamic and gas exchange variables before and 1 hour after blood transfusion are presented in Table 2. Hb and hematocrit increased after blood transfusion in both groups (*P* <0.01). After blood transfusion, a decrease in base excess was found only in the non-leukodepleted group (*P* <0.05). Baseline MAP and PaO_2_/FiO_2_ were lower in the non-leukodepleted group compared with the leukodepleted group. MAP increased after transfusion only in the non-leukodepleted group (*P* = 0.04). No other significant differences between groups or time points were found.

**Table 2:** Hematologic, hemodynamic, and gas exchange variables and Sequential Organ Failure Assessment score (baseline and 1 hour after blood transfusion)

	**Non-leukodepleted group**			**Leukodepleted group**		
	**n = 10**			**n = 10**		
	**Before**	**After**	***P***^**a**^	**Before**	**After**	***P***^**a**^
Hb, g/dL	8.4 (7.9–8.8)	10.4 (9.9–11.5)	<0.01	8.3 (7.4–8.6)	10.4 (9.9–10.8)	<0.01
Hct, %	26.7 (26.0–28.0)	32.5 (29.9–34.6)	<0.01	25.9 (22.9–27.9)	31.5 (29.5–33.5)	<0.01
HR, bpm	72 (59–98)	70 (60–86)	0.20	91 (79–97)	88 (81–94)	0.60
MAP, mm Hg	70 (67–77)	77 (72–98)	0.04	85 (75–106)^b^	89 (78–96)	0.68
Urine output, mL/d	3,145 (2,516–3,625)	3,572 (2,385–4,266)	0.99	3,520 (2,756–4,854)	4,636 (2,967–5,314)	0.07
T, °C	36.8 (35.9–37.3)	36.7 (36.1–37.4)	0.48	36.9 (36.5–37.4)	37.4 (36.5–37.9)	0.12
WBCs, ×10^3^/μL	11.9 (5.2–17.3)	12.5 (5.1–17.2)	0.99	8.7 (5.9–15.4)	8.6 (6.6–13.3)	0.76
PLTs, ×10^3^/μL	190 (112–225)	198 (87–216)	0.86	163 (90–336)	172 (100–332)	0.27
pH	7.48 (7.36–7.54)	7.49 (7.37–7.52)	0.07	7.48 (7.46–7.5)	7.48 (7.47–7.50)	0.99
PaO_2_, mm Hg	123 (103–149)	105 (96–125)	0.06	133 (94–183)	137 (119–153)	0.41
PaCO_2_, mm Hg	42 (36–45)	42 (37–46)	0.94	39 (34–41)	39 (35–41)	0.76
PaO_2_/FiO_2_	230 (206–309)	215 (173–298)	0.36	323 (243–408)^b^	287 (270–374)	0.34
BE, mEq/L	5.7 (1.8–10.5)	5.3 (1.3–9.5)	0.03	3.7 (2.8–5.9)	3.9 (2.2–5.8)	0.73
Lac, mmol/L	1.2 (0.9–1.7)	1.3 (1.0–1.8)	0.44	1 (0.7–1.4)	1.1 (0.7–1.4)	0.53
SOFA score	8 (5–12)	8 (3.4–12)	0.85	5 (3–7)	5 (3–8)	0.59

^a^Pre versus post, Wilcoxon matched–pairs signed–rank test. ^b^*P* <0.05, versus non–leukodepleted red blood cell group at the same time point, Mann–Whitney *U* test. BE, base excess; FiO_2_, fraction of inspired oxygen; Hb, hemoglobin; Hct, hematocrit; HR, heart rate; Lac, arterial lactate levels; MAP, mean arterial pressure; PaCO_2_, arterial partial pressure of carbon dioxide; PaO_2_, arterial partial pressure of oxygen; PLT, platelet; SOFA, Sequential Organ Failure Assessment; T, body temperature; WBC, white blood cell.

### Sidestream dark field- and near-infrared spectroscopy-derived variables

**Figure 1:**
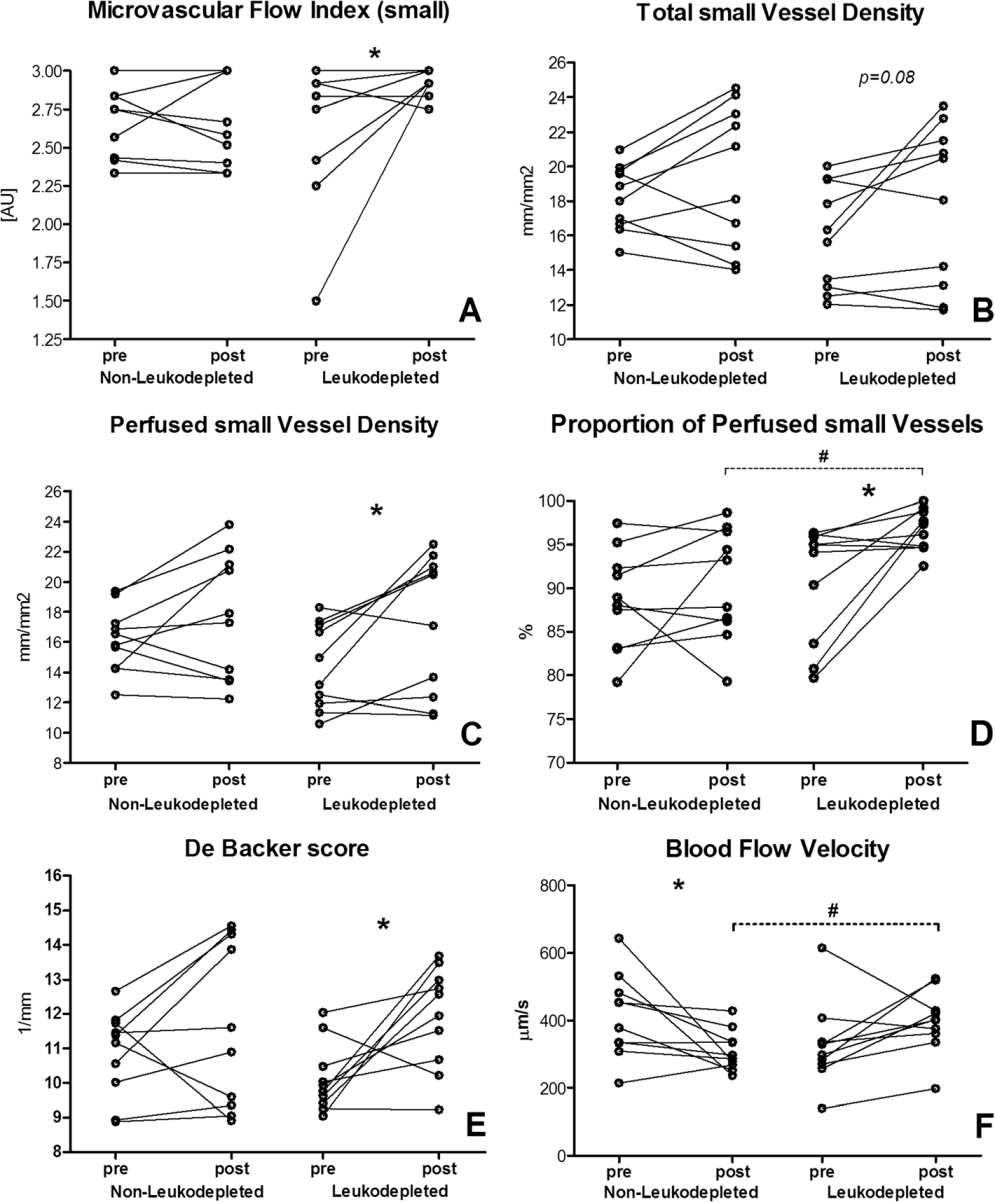
**Individual changes in microcirculatory parameters after blood transfusion in non-leukodepleted and leukodepleted groups. (A)** Microcirculatory flow index (in small vessels). **(B)** Total small vessel density. **(C)** Perfused small vessel density. **(D)** Proportion of perfused small vessels. **(E)** De Backer score. **(F)** Blood flow velocity. *P <0.05, Wilcoxon matched-pairs signed-rank test; ^#^*P* <0.05, Mann-Whitney *U* test.

Microcirculatory and near-infrared spectroscopy (NIRS)-derived variables before and 1 hour after blood transfusion are presented in Table 3. When groups were compared, at baseline there were no statistically significant differences, but after transfusion, higher PPV and blood flow velocity were observed in the leukodepleted group (Figure 1). Concerning the changes between before and after transfusion, MFI and blood flow velocity exhibited different responses—MFI: −0.02 (−0.1 to 0.04) in the non-leukodepleted group and 0.17 (0 to 0.54) in the leukodepleted group, *P* = 0.03; blood flow velocity: −56 (−183 to 16) in the non-leukodepleted group and 68 (11 to 170) in the leukodepleted group, *P* = 0.04. When looking at within-group changes, compared with baseline, MFI, PVDs, PPV, and De Backer score increased in patients who received leukodepleted RBCs but not in those who received non-leukodepleted RBCs (Figure 1). Blood flow velocity decreased only in the non-leukodepleted group (Figure 1). No correlation was found between changes in microvascular parameters and MAP after blood transfusion. The change in PPV after blood transfusion was negatively correlated with baseline PPV in the leukodepleted group (*r* = −0.72, *P* = 0.02); this relationship was lacking in the non-leukodepleted group (*r* = −0.36, *P* = 0.3).

**Table 3:** Microcirculatory parameters, near-infrared spectroscopy-derived variables, and glycocalyx variables (baseline and 1 hour after blood transfusion)

	**Non-leukodepleted group**			**Leukodepleted group**		
	**n = 10**			**n = 10**		
	**Before**	**After**	***P***^**a**^	**Before**	**After**	***P***^**a**^
MFI, AU	2.75 (2.43–2.87)	2.62 (2.38–3.00)	0.73	2.79 (2.37–2.92)	2.96 (2.89–3.00)	0.04
De Backer score, 1/mm	11.3 (9.7–11.8)	11.3 (9.3–14.4)	0.16	9.8 (9.4–10.8)	12.3 (10.6–13.1)	0.02
TVD, mm/mm^2^	18.4 (16.6–19.8)	19.6 (15.1–23.3)	0.19	15.9 (12.9–19.3)	19.3 (12.8–21.8)	0.08
PVD, mm/mm^2^	16.2 (14.3–17.7)	17.6 (13.5–21.4)	0.23	14.1 (11.8–17.2)	18.8 (12.1–21.2)	0.04
PPV, %	88.5 (83.1–93.1)	90.6 (85.8–96.6)	0.32	94.6 (82.9–95.9)	96.8 (94.7–98.9)^b^	0.01
HI	0.23 (0.13–0.50)	0.16 (0.00–0.32)	0.31	0.13 (0.06–0.23)	0.04 (0.00–0.10)	0.11
Blood flow velocity, μm/s	415 (327–494)	291 (264–314)	0.03	314 (267–354)	402 (355–451)^b^	0.08
THI, AU	10.5 (7.8–11.2)	13.4 (10.4–15.8)	<0.01	10.3 (8.6–13.6)	13.8 (10.6–15.7)	0.02
StO_2_, %	88 (80–90)	90 (85–93)	0.03	83 (77–92)	86 (82–89)	0.59
StO_2_ down, %/min	−9.5 (−11 to −8.5)	−9.0 (−10.4 to −7.5)	0.03	−10.1 (−12.8 to −6.9)	−9.2 (−10.4 to −7.6)	0.56
StO_2_ up, %/min	173.6 (81.26–220.4)	191.3 (133.4–242.4)	0.01	197.9 (99.2–260.3)	206.2 (131.1–291.8)	0.03
AUC StO_2_, %×min	10.7 (8.4–21)	10.9 (8.2–25.4)	0.99	8.0 (4.4–13.8)	12.1 (5.5–18.3)	0.51
PBR, μm	2.69 (2.53–2.94)	2.72 (2.65–2.86)	0.23	2.80 (2.76–2.97)	2.74 (2.58–3.00)	0.73
Syndecan–1, ng/mL	219.4 (84.5–361.5)	310.1 (80.9–498.8)	0.03	100.0 (5.4–378.4)	127.5 (6.6–505.6)	0.37
Heparan sulfate, ng/mL	23.3 (19.7–39.5)	44.7 (9.1–79.2)	0.43	63.1 (40.5–96.0)^b^	48.7 (39.0–79.2)	0.37
Hyaluronan, ng/mL	211.9 (75–423.7)	198.1 (94.2–320.0)	0.37	275.7 (109.3–450.7)	249.4 (129.2–433.3)	0.32

^a^Pre versus post, Wilcoxon matched–pairs signed–rank test. ^b^*P* <0.05, versus non–leukodepleted red blood cell group at the same time point, Mann–Whitney *U* test. Microvascular flow index (MFI), total vessel density (TVD), perfused vessel density (PVD), proportion of perfused vessels (PPV), and flow heterogeneity index (HI) were calculated in small vessels (diameter <20 μm). AUC StO_2_, area under the curve of the tissue oxygen saturation (reactive hyperemia following the vascular occlusion test); PBR, perfused boundary region; StO_2_, tissue oxygen saturation; StO_2_ down, tissue oxygen saturation downslope (ischemic phase of the vascular occlusion test); StO_2_ up, tissue oxygen saturation upslope (reperfusion phase of the vascular occlusion test); THI, tissue hemoglobin index.

**Figure 2:**
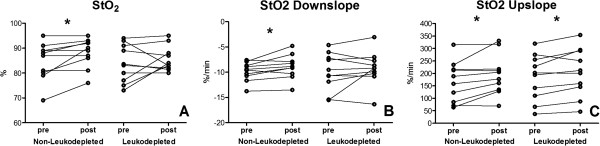
**Individual changes in near-infrared spectroscopy (NIRS)-derived variables after blood transfusion in non-leukodepleted and leukodepleted groups. (A)** Tissue oxygen saturation (StO_2_) baseline. **(B)** StO_2_ downslope (ischemic phase during the vascular occlusion test). **(C)** StO_2_ upslope (reperfusion phase during the vascular ocllusion test). *P <0.05, Wilcoxon matched-pairs signed-rank test.

No significant differences were observed in NIRS-derived parameters at baseline and after transfusion. In regard to within-group changes, StO_2_ upslope and THI were elevated in both groups (Figure 2C). Baseline StO_2_ (Figure 2A) and StO_2_ downslope (Figure 2B) increased in the non-leukodepleted group. No difference in the AUC for StO_2_ was found in either the between-group or the within-group analysis.

### Glycocalyx measurements

**Figure 3:**
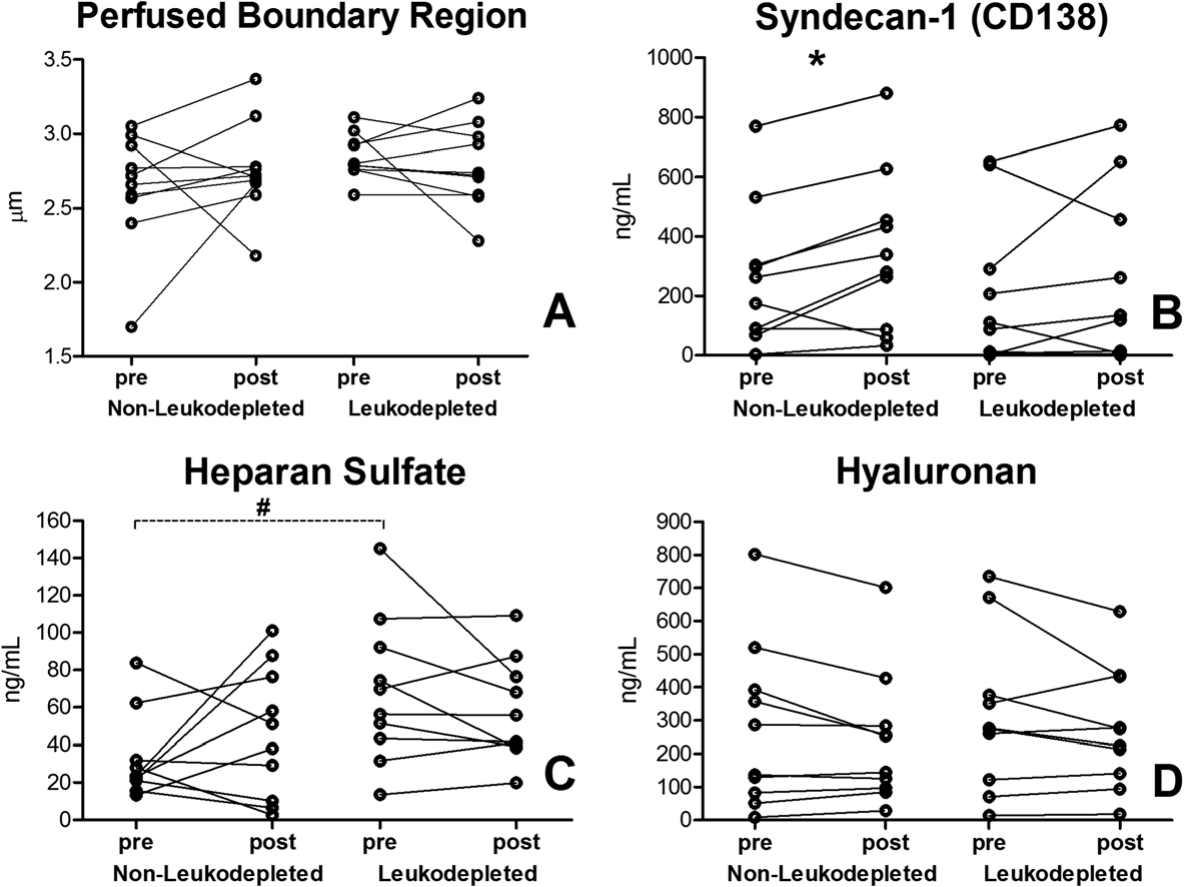
**Effects of the transfusion of non-leukodepleted and leukodepleted red blood cells (RBCs) on the endothelial glycocalyx. (A)** Perfused boundary region. **(B)** Syndecan-1. **(C)** Heparan sulfate. **(D)** Hyaluronan. *P <0.05, Wilcoxon matched-pairs signed-rank test; ^#^*P* <0.05, Mann-Whitney *U* test.

**Figure 4:**
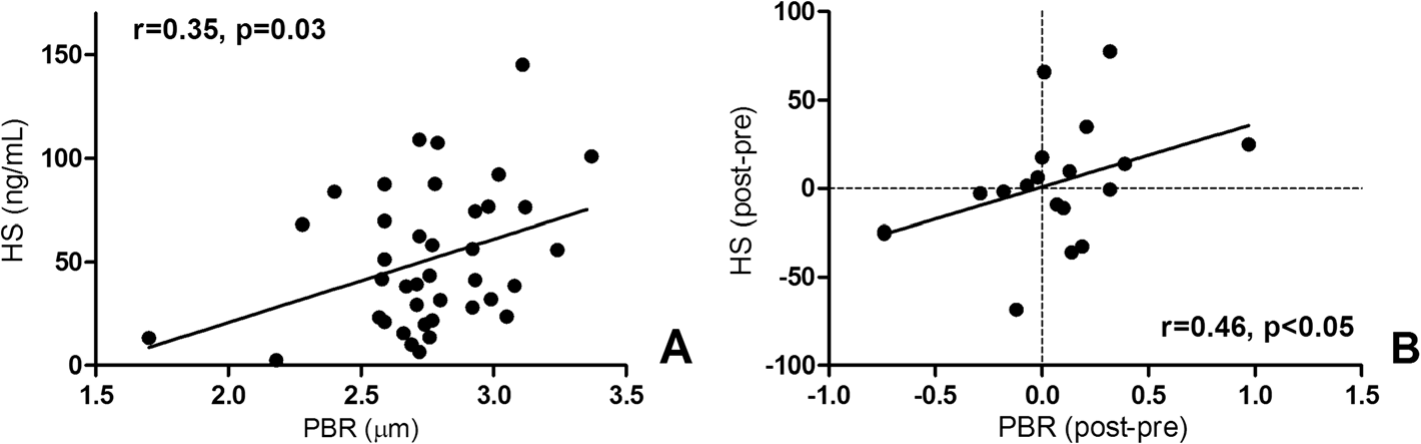
**Correlation between perfused boundary region (PBR) and serum heparan sulfate (HS). (A)** Correlation between all PBR values and serum HS values. **(B)** Correlation between changes in PBR and serum HS after blood transfusion.

Baseline heparan sulfate was higher in the leukodepleted group, and no significant differences were observed for baseline syndecan-1, hyaluronan, and PBR between the groups. After blood transfusion, PBR, heparan sulfate, and hyaluronan did not change between the two groups (Figure 3). Syndecan-1 increased after the transfusion of non-leukodepleted RBCs (Figure 3B). A minor correlation was found between PBR values and heparan sulfate levels (*r* = 0.35, *P* = 0.03) as well as between PBR changes and heparan sulfate changes after blood transfusion in the whole sample (*r* = 0.46, *P* = 0.04) (Figure 4). No correlation was found between PBR values and syndecan-1 or hyaluronan levels.

## Discussion

The present study does not show a clear superiority of leukodepleted over non-leukodepleted RBC transfusion on microvascular perfusion in patients with sepsis, but the more favorable changes observed in MFI and blood flow velocity suggest a positive effect of leukodepleted blood transfusion on microcirculatory convective flow. The within-group analysis indicates that patients who received leukodepleted RBCs exhibited a more consistent overall improvement in the microvascular parameters. The lack of further differences in the between-group comparisons may just reflect that the study was underpowered.

The sublingual microcirculation is used as a model to study and extrapolate information representing splanchnic blood flow. Persistent microvascular alterations are associated with the occurrence of organ failures and death in patients with septic shock [37]. Previous studies showed that blood transfusions were not able to reverse microcirculatory hypoperfusion in patients with severe sepsis [20,22]. However, the characteristics of the transfused RBCs, in terms of storage and leukocyte reduction, were not examined in previous investigations. The present study did not demonstrate a clear advantage of leukodepleted over non-leukodepleted RBC transfusion on the microcirculation. Indeed, the sublingual microvascular parameters were mostly not significantly different after transfusion between the two groups, and the difference in PPV might totally depend on the basal disparity. Nevertheless, the MFI, which was the primary endpoint of the study, showed significantly different changes after the transfusion of leukodepleted versus non-leukodepleted RBC units. Similarly, different effects were observed through the quantification of blood flow velocity, which was higher after the transfusion of leukodepleted RBCs. These results would suggest a beneficial effect of leukodepleted blood transfusion on the convective flow in the microcirculation. Lower adhesiveness to vascular endothelial cells has been reported for leukodepleted compared with non-leukodepleted and buffy coat-poor blood [38,39] and may account for a better hemorrheological impact of leukodepleted RBCs. In addition, the within-group analysis indicated more consistent improvements in several microvascular parameters in patients who received leukodepleted RBCs, as represented by increased microvascular density and percentage of perfused vessels. The transfusion of non-leukodepleted RBCs did not yield any notable improvement in the sublingual microcirculation; a reduction in blood flow velocity was observed. Nevertheless, several points should be considered. First, the responses observed may depend not only on the type of transfused RBCs but also on the underlying clinical and microvascular status. We studied a heterogeneous population by including patients with sepsis, severe sepsis, or septic shock. Second, the two groups were not adequately balanced; in fact, the patients in the non-leukodepleted group appeared to be more severely ill in general as indicated by the lower MAP and PaO_2_/FiO_2_, higher SOFA score (even if not significant), and higher proportion of patients with septic shock. Therefore, definite conclusions cannot be extrapolated from the results of the present study, as the meaning of any comparison between the groups remains uncertain. Notably, however, one would have expected the more severely ill patients in the non-leukodepleted group to show bigger microvascular improvements: indeed, the microcirculatory response to blood transfusion demonstrated a negative correlation with the baseline status in patients with severe sepsis [20]. In our study, the increase in PPV was inversely related to baseline values in patients who received leukodepleted RBCs; interestingly, this correlation was lacking in the non-leukodepleted group. Finally, most patients were studied several days after their ICU admittance and were already hemodynamically stable, as reflected by their low SOFA score, normal heart rate, low arterial lactate levels, and absence of metabolic acidosis. Conversely, metabolic alkalosis was seen in both groups; the most plausible reason was that 17 patients out of 20 had been treated with furosemide for some days before their inclusion in the study. Moreover, most patients did not show big microcirculatory alterations at baseline; PPV was above 75% in all patients, and the median MFI was above 2.6 in both groups [30]. This may have conditioned the response observed. The transfusion of leukodepleted RBCs was able to improve microvascular perfusion and tissue oxygenation in patients with a relatively healthy microcirculation [40,41] but did not show any significant effect in severely septic patients with dysfunctional microcirculation [20]. Therefore, our findings could not be extended to septic patients with severe microvascular derangement. Moreover, it remains a matter of debate whether most of our patients with an already-resuscitated microcirculation really required an increase in microvascular perfusion and whether such an increase could have any beneficial impact on outcome.

We found an increase in serum syndecan-1 levels after the transfusion of non-leukodepleted RBCs, suggesting fragmentation of the endothelial glycocalyx. The glycocalyx is believed to fulfil an important role in maintaining microvascular hemorrheological homeostasis. Interestingly however, it can be easily damaged by oxidative stress and inflammatory mediators [36,42,43]. We did not find any change in hyaluronan, heparan sulfate, and PBR in either group, nor did we find differences in these parameters after blood transfusion between the groups. Therefore, our results do not allow any conclusion on the effect of the transfusion of the two types of RBCs on the glycocalyx. Large variability was seen in the baseline glycocalyx status in the studied patients; perhaps in relation to the severity of sepsis, this heterogeneity may have produced different responses to blood transfusion.

The transfusion of banked leukodepleted RBCs, stored up to 42 days, showed no substantial effect on muscle StO_2_ and microvascular reactivity in critically ill patients [21]; similar results were found in severely septic patients with the transfusion of non-leukodepleted RBCs stored up to 42 days [22]. Blood transfusions increase blood viscosity [41], thus leading to shear stress-induced vasodilation by nitric oxide production [44]. During hypoxia, RBCs can release nitric oxide and ATP, thereby exerting a direct vasodilator effect [21]; these properties may be impaired during prolonged storage [4,5]. In the present study, microvascular reactivity improved after blood transfusion in both groups, as reflected by the increase in NIRS-derived StO_2_ upslope during the reperfusion phase of the VOT. We could speculate that the transfusion of fresh RBCs with preserved hemorrheological properties was responsible for the observed effect. Nevertheless, the discrepancy between our results and previous findings might reasonably be explained also by differences in the studied patient populations.

Despite the increase in THI in both groups, StO_2_ and StO_2_ downslope increased only in the non-leukodepleted group. It should be noted that similar absolute change, though not significant, was observed in the leukodepleted group. We acknowledge that this study may have been underpowered to detect changes in these parameters. An increase in muscle and sublingual tissue oxygenation was reported after the transfusion of leukodepleted RBCs in hematology outpatients [41]. On the contrary, previous studies in critically ill and severely septic patients did not show any effect of blood transfusion on tissue oxygenation measured by the NIRS technique [21,22]. Substantial differences in the baseline microvascular status may again explain these discrepant results.

The transfusion of leukodepleted RBCs has been associated with reduced hospital length of stay [45], incidence of infections [46], transfusion-related acute lung injury [47], and acute kidney injury [48] in various populations of patients. It has been reported that transfusion of leukodepleted RBCs is not associated with increased mortality in patients with septic shock [49]. It has been demonstrated that pre-storage leukoreduction can prevent the accumulation of cytokines and other inflammatory mediators from residual leukocytes, thereby avoiding potentially detrimental effects in the recipient [50-52]. All of these findings derived from studies using pre-storage leukoreduction. Notably, in our study, RBC units underwent a post-storage leukoreduction before transfusion. This procedure does not prevent the accumulation of leukocyte-derived cytokines during the first days of storage. This might be one of the reasons why we found only slight differences in the microcirculation after blood transfusion between the two groups. Unfortunately, we could not investigate the potential advantages of pre-storage leukoreduction, since this is not usually performed in our blood bank. Nevertheless, microvascular improvement observed in the leukodepleted group might also suggest that post-storage leukoreduction can still prevent deleterious effects of the transfusion of RBC units containing allogenic leukocytes. Further studies comparing pre- and post-storage leukocyte reduction would be needed to clarify this point.

Our study has several limitations that should be considered when extrapolating the data reported in this clinical investigation. First, the low number of patients enrolled may have resulted in underpowered statistical analysis, and differences in some variables may not have been detected. Second, the inclusion of patients with different severity of sepsis could have influenced the microvascular responses to blood transfusion. Unfortunately, the small sample size did not allow a *post hoc* analysis in order to distinguish between sepsis, severe sepsis, and septic shock subgroups. Our study was designed to include a heterogeneous population of patients with different severity of sepsis. Future studies should be focused on more homogeneous subgroups of patients with sepsis. Third, the baseline differences observed between non-leukodepleted and leukodepleted groups prevented a proper between-group comparison; in fact, we acknowledge that our results are based mostly on a within-group analysis which cannot reliably support the benefit of leukodepleted over non-leukodepleted RBCs in patients with sepsis. Finally, the inclusion of stable septic patients with an already-restored microvascular perfusion may represent a limitation of our study; patients with a dysfunctional microcirculation may have revealed a different response to the same blood transfusions. This point should be addressed in future studies. Despite these limitations, our study remains interesting as it provides a comprehensive evaluation of microcirculatory responses to blood transfusion in a heterogeneous population of patients with sepsis. As a pilot study on a small number of patients, our investigation cannot provide conclusive answers, nor was it aimed at this goal. Our objective was to explore whether the transfusion of leukodepleted RBCs could provide any advantage on the microcirculation in patients with sepsis. Adequately powered studies should be performed in order to better define the potential benefits observed.

## Conclusions

In this pilot study, we were not able to demonstrate a clear benefit of leukodepleted over non-leukodepleted RBC transfusion on the microcirculation in patients with sepsis. However, our results suggest a more favorable effect of leukodepleted RBCs on microcirculatory convective flow. In addition, the within-group analysis showed more consistent improvements in several microvascular parameters in patients who received leukodepleted RBCs. The lack of further differences in the between-group comparisons may just reflect that the study was underpowered. Further studies are needed to confirm these findings.

## Key messages

•No clear difference was found in most microvascular parameters after the transfusion of leukodepleted or non-leukodepleted red blood cells (RBCs) in our small and heterogeneous population of patients with sepsis.

•The transfusion of leukodepleted RBCs compared with non-leukodepleted RBCs showed a more favorable effect on microvascular flow index and blood flow velocity, suggesting a benefit on microcirculatory convective flow.

•In the within-group analysis, an overall microvascular improvement was seen only in patients who received leukodepleted RBC transfusion.

## Abbreviations

AOU: Azienda Ospedaliero Universitaria; AUC: area under the curve; ELISA: enzyme-linked immunosorbent assay; FiO2: fraction of inspired oxygen; Hb: hemoglobin; ICU: intensive care unit; IL: interleukin; MFI: microvascular flow index; NIRS: near-infrared spectroscopy; paO2: arterial partial pressure of oxygen; PBR: perfused boundary region; PPV: proportion of perfused vessels; PVD: perfused vessel density; RBC: red blood cell; SDF: sidestream dark field; SOFA: Sequential Organ Failure Assessment; StO2: tissue oxygen saturation; THI: tissue hemoglobin index; VOT: vascular occlusion test.

## Competing interests

CI is the inventor of SDF imaging technology and holds shares in MicroVision Medical and was a consultant for this company more than four years ago but has had no further contact with the company since then. He declares that he has no other competing interests in this field other than his commitment to promoting the importance of the microcirculation during patient care and no other relationships or activities that could appear to have influenced the submitted work. HV holds the position of chief scientific officer in GlycoCheck BV. The other authors declare that they have no competing interests.

## Authors' contributions

AD designed the study, performed the statistical analysis, drafted the manuscript, and interpreted the data. ED, RD, CS, AC, VG, PC, EA, and HV made substantial contributions to the acquisition of the data and the analysis of SDF videos and revised the manuscript for important intellectual content. MML and AG performed immunoenzymatic assays and helped to draft the manuscript. RB performed leukoreduction, randomly provided the type of RBCs, and helped revise the manuscript for important intellectual content. MP and PP supervised and evaluated the study, helped in the analysis of the data, and revised the manuscript. CI made a substantial contribution in the study design and interpretation of the data and critically revised the manuscript for important intellectual content. All authors had full access to the data, take responsibility for the integrity of the data and the accuracy of the analysis, and have read and approved the final manuscript.
